# A Case of Successful Amputation at the Shoulder Joint in Consequence of an Injury Sustained by the Patient Fifteen Years Ago

**Published:** 1849-08

**Authors:** Wm. Byrd Page

**Affiliations:** Consulting Surgeon to the Philadelphia Hospital, (Blockley,) Lecturer on Obstetrics in the Medical Institute, &c.


MEDICAL EXAMINER,

AND

RECORD OF MEDICAL SCIENCE.

NEW SERIES.—NO. LVI.—AUGUST, 1849.

### ORIGINAL COMMUNICATIONS.

*A Case of successful Amputation at the Shoulder Joint in consequence of an injury sustained by the patient fifteen years ago.* By Wm. Byrd Page, M. D., Consulting Surgeon to the Philadelphia Hospital, (Blockley,) Lecturer on Obstetrics in the Medical Institute, &c.

I was called, about 1st December, 1849, by Dr. Elwood Wilson to see Michael Byrne, aged 56 years, who had been for several weeks under treatment for a severe inflammation in the neighborhood of the right shoulder joint.

Michael stated that, about fifteen years before, when in Ireland, while leading a young horse, he was suddenly thrown down and dragged for some distance. His right shoulder and left hip were seriously injured, though the nature of his accident did not seem to have been well understood. He suffered much at the time, and placed himself in the hands of a surgeon, from whose treatment he says that he derived very little benefit. He states that a discharge of matter has occasionally taken place from the neighborhood of the shoulder after an attack of inflammation, and that a number of small pieces of bone have been discharged at different times. His arm has been almost powerless since his injury, hanging generally a useless appendage by his side, unless supported in a sling.

He attributes his present attack to an attempt at sawing wood, by which he jarred his shoulder, and gave rise to inflammation. On a careful examination of the shoulder, a profuse suppuration was discovered to have taken place, which extended to a considerable distance, and which gave rise to great pain in consequence of the enormous distension of the parts. The patient's strength was fast failing under the suppuration, and the attendant constitutional disturbance. He had been for some time unable to sleep, was very nervous, had no appetite, was bathed in perspiration, and was suffering constant and severe pain in the shoulder and arm.

I at once punctured the abscess in the most prominent point below and in front of the acromion process, and allowed a large quantity of fœtid ill-conditioned greenish pus to escape. On the introduction of the probe, the cavity of the abscess was found to be very large, and the upper portion of the humerus to be rough and bare, while some loose bone could be felt. Stimulants, opiates and good diet were resorted to, and a large flaxseed poultice was applied to the shoulder. The patient's condition improved materially in the course of a few days, and the tumefaction of the parts so far subsided as to allow us to make a more satisfactory examination. A hard, round tumour, supposed to be the head of the humerus, was distinctly felt beneath the clavicle, and was immoveable. The arm was very moveable and entirely powerless, having no connexion with the tumour under the clavicle. The parts were still so altered that the precise anatomical connexion could not be made out, but enough was detected to show that an irreparable lesion existed about the joint, that the upper portion of the humerus was diseased, and that the patient must soon be worn out by the discharge, which continued to be very great. On further consultation, in which Dr. Peace participated, it was determined that amputation at the shoulder joint would alone afford him a chance for relief. He, however, could not make up his mind to the loss of his arm until he became more fully aware of his dangerous condition. Several openings were made, and others occurred spontaneously about the shoulder along the arm, and on the side of the chest below the axilla, to which region the abscess ultimately extended. He was carefully watched, and supported by stimulants, tonics and good diet, until the 26th December, when a profuse hemorrhage took place from the openings into the sac, which exhausted him to the last degree, and which was arrested with much difficulty, by plugging up the outlets with lint, and the application of graduated compresses, which were secured by a bandage. Being very much prostrated, and fearing that he would die, he now made up his mind to have his arm removed, should his condition again become suitable.

With little hope of saving his life, a supporting treatment was fully pursued until the 16th January, 1849, when the operation, for which he had become anxious, was decided upon.

With the advice and assistance of Drs. E. Wilson, Seymour, Spenser Sergeant, D. H. Tucker and Peace, I proceeded to the performance of the operation.

The patient, after taking a dose of brandy and laudanum was placed on a bed, with his shoulder resting over its side. The arm was held by Dr. Tucker, and the subclavian artery was well secured by Dr. Sergeant by pressure with a key on its passage over the rib. The shoulder was pierced with a long double edged knife, which was introduced in front of, and a little below, the acromion process of the scapula, and the upper flap made by cutting as closely as possible to the humerus down below the insertion of the deltoid. The knife was then carried around the upper extremity of the bone, and along its under surface to a point opposite the termination of the superior flap, and the limb removed. The upper incision was somewhat interfered with by the instruments coming in contact with a large piece of loose bone. The axillary, and other large and many small arteries, were speedily secured without the loss of much blood.

A portion of the head of the humerus was found to be firmly united to the second rib, the superior part of which was carious. It became a question, whether the whole of this portion of the bone should be removed, but as the union to the rib was very firm, and as the neighboring parts were very vascular, it was determined to remove the diseased portions alone, which was accomplished, after its detachment from the surrounding muscle, by the use of Liston's large bone nippers.

The glenoid cavity with its cartilaginous lining and margin was found to be entirely unoccupied, with the synovial membrane smooth and healthy, which is altogether in opposition to the statement of surgical writers, that a cavity from which the head of a bone has been removed for some time becomes ultimately filled up. No new socket was found, nor had the old one disappeared.

A single stitch was placed in the upper angle of the wound, and the parts were brought together by adhesive strips, and the usual dressings applied.

The operation was borne much better than was anticipated, and the patient expressed himself quite comfortable after being put to bed. Brandy and water with laudanum was administered and gruel allowed.

Five hours after the operation a profuse hemorrhage occurred, which rendered it necessary to open the wound. Several small vessels were tied, and tannic acid was freely applied to the angle of the wound, high up between the remnant of the head of the humerus and the clavicle, from which point much blood was lost. The patient was very much exhausted by the hemorrhage, and complained so much of pain in the parts that I was induced to bring the edges of the wound very imperfectly together.

For several days he was in great danger, and was kept alive by active stimulation. Carbonate of ammonia, opium and camphor, with punch, brandy, porter and quinine were administered in as large doses as could be borne, and we had the satisfaction of finding our patient gradually recovering under their use. From having been constantly in bed for a long time in a helpless condition, bed sores began to form, which were successfully treated by change of position, the use of small cushions, frictions with spirits of camphor, and the application of soap plaster. On the 18th day after the operation, the ligature separated from the axillary artery, the others having been removed from day to day.

The edges of the wound when in apposition, healed by the first intention, but in consequence of their separation, especially at the upper and anterior portion, a good deal of the cure was effected by granulation, which was assisted by the application of Turner's cerate and a solution of quinine and sulphate of copper. A considerable sac existed in the wound, partly formed by the glenoid cavity, and partly by the ununited surfaces of the upper portion of the flaps. This was caused to be filled up by daily injections of sulphate of copper and quinine, alternated with sulphate of iron and quinine, the solutions being in the proportion of ten grains of each ingredient to the ounce of water. Wet and dry dressings were used throughout the cure according to circumstances. A bandage was firmly applied around the chest, which, with the use of the above injections, caused the sides of the abscess below the axilla to unite. The internal medication consisted im the above mentioned stimulants, tonics, and anodynes, with mild aperients to keep the bowels open, which were very inactive. A troublesome heart-burn was relieved by an infusion of gentian and super carb. of soda, alternated with a mixture of baker's yeast and effervescing draught. A highly nutritious diet was persisted in.

In about six weeks, Michael was able to sit up and walk about his own room, though suffering from debility and swelling of his legs. His strength has gradually returned to him, and he has been for some time going about the streets, with a locomotion not altogether perfect, in consequence of the old injury to his left hip. He has still a slight discharge from the upper portion of the wound, with which I have thought it best not to interfere, as it is very trifling, and perhaps serves a salutary purpose.

Yesterday, June 22, 1849, I saw Michael. He is fat and hearty, is cheerful, is perfectly satisfied with the result of the operation, and is especially content that he is still in the land of the living.

My thanks are due to the gentlemen who assisted in the operation, and especially to Drs. Wilson and Seymour, as on their unremitting and assiduous attentions during the whole treatment of the case, did its success depend.

